# The increasing burden of alcohol-related traumatic brain injury in older adults – Two decades of population-based data from Southwestern Norway

**DOI:** 10.1016/j.bas.2026.106121

**Published:** 2026-06-10

**Authors:** Mustafa Nafi Al-Falah, David Andreas Thomas Werner, Kjell Alexander Thunes Akre, Kenneth Thorsen, Martine A. Aarsland, Arezo Kanani, Clemens Weber

**Affiliations:** a Faculty of Medicine, University of Bergen, Bergen, Norway; b Department of Neurosurgery, Stavanger University Hospital, Stavanger, Norway; c Department of Gastrointestinal Surgery, Stavanger University Hospital, Stavanger, Norway; d Stavanger Trauma Investigation Group, Stavanger University Hospital, Stavanger, Norway; e Department of Orthopedic Surgery, Stavanger University Hospital, Stavanger, Norway; f Department of Quality and Health Technology, The Faculty of Health Sciences, University of Stavanger, Stavanger, Norway

**Keywords:** Epidemiology, Traumatic brain injury, Alcohol, Elderly

## Abstract

**Objective:**

The objective of this study was to assess epidemiological patterns of alcohol-related traumatic brain injury (TBI) in older adults over a period of 20 years.

**Methods:**

This is an observational population-based study utilizing prospectively collected data from the institutional trauma registry at Stavanger University Hospital in Southwestern Norway. Patients aged 60 years and older admitted due to TBI between January 1, 2004, and December 31, 2023, were included. Patient demographics and trauma-related variables, and outcome after TBI were described.

**Results:**

A total of 1021 older patients with TBI were admitted during the 20 year-study period; 172 (17%) were under the influence of alcohol. Most patients suffered mild TBI (73%), and fall related injuries were the most common mechanism of injury (76%). The crude incidence rate increased from 40 to 81 TBI cases per 100 000 population yearly. Alcohol-related TBI tripled, while non-alcohol-related cases doubled during the study period. The overall 30-day mortality rate was 16%.

**Conclusion:**

This study shows an increasing number of hospital-registered TBI in older patients, with also increasing alcohol-related TBI in this age group. The triad of aging, alcohol consumption, and TBI represents an essential future challenge, requiring focused efforts and evidence-based interventions to reduce both human and socio-economic burdens.

## Highlights

• In this long-term study over 20 years, TBI is increasing in elderly populations.
• The number of alcohol-related TBI patients increases more than non-alcohol-related TBI in elderly patients.
• Elderly men are overrepresented in both the total cohort and the alcohol-related TBI subgroup.

## 1 Introduction

Traumatic brain injury (TBI) represents a common and escalating worldwide health burden, with an estimated incidence of more than 50 million patients per year (Zhong et al., 2025). The two most predominant causes of TBI are traffic accidents, which show a declining trend, and low-energy falls, whose frequency is increasing due to an aging population, especially in high-income countries (Andreassen et al., 2022). Older patients demonstrate substantially higher rates of TBI related to low-energy falls compared to younger patients with high mortality and morbidity rates (Francis et al., 2025).

The causes of falls among older patients are complex and multifactorial, including age-related changes in body composition, comorbidity, and polypharmacy, which subsequently impair muscular strength, cognitive function, postural balance, and motor coordination (Pillay et al., 2024). Furthermore, additional risk factors contribute significantly, including behavior and lifestyle.

Alcohol is a well-recognized behavioral determinant in the context of trauma (Chikritzhs and Livingston, 2021). Several studies, particularly those focusing on older patients, demonstrate a negative effect of alcohol, and with a reduced reserve capacity, chronic diseases, and polypharmacy, the tolerance of alcohol is even more decreased (national institute on alcohol abuse and alcoholism, 2024). Current research suggests that older patients consume more alcohol compared to previous generations (Breslow et al., 2017; Tevik et al., 2019). This is therefore an emerging critical challenge in future healthcare systems dominated by an older population where well-defined and early strategies within prevention, diagnosis, treatment and rehabilitation are essential. However, there is a lack of studies that address the intersection of TBI, older patient populations and alcohol use.

This study aims to evaluate the epidemiology of alcohol-related TBI in older adults over a period of 20 years, including its proportion, injury mechanisms, temporal trends, and clinical outcomes.

## 2 Methods

### 2.1 Study design and period

This study is an observational population-based study utilizing prospectively collected data from the institutional trauma registry at Stavanger University Hospital (SUH) in Norway. The study adheres to the STROBE guidelines for observational research (von Elm et al., 2014). The study timeframe spans two decades, from January 1, 2004, to December 31, 2023. In compliance with ethical standards, the present observational study obtained approval from The Regional Committee for Medical and Health Research Ethics of Western Norway (REC-ID 143902).

### 2.2 Study population and collected data

The study cohort comprised patients aged 60 years and older with a confirmed diagnosis of TBI recorded in the institutional trauma registry at Stavanger University Hospital, a local trauma center delivering acute trauma care, including neurosurgery. The Norwegian health care system is based on regional referral and SUH is the only hospital in the southwestern region of Norway receiving and admitting patients with traumatic brain injury (TBI) (Heskestad et al., 2009). The hospital manages trauma cases from a wider population of about 550.000 inhabitants (Wiik-Larsen et al., 2020).

The trauma registry includes hospitalized patients with traumatic injuries according to predefined institutional inclusion criteria. All patients required trauma team activation were automatically registered regardless of injury severity (AIS 1–6). Furthermore, all admitted TBI patients with an AIS severity score ≥3 are manually registered by certified registars following assessing radiographic and clinical records. Isolated mild TBI (AIS 1-2) and external face and head injuries are excluded unless associated with multiple anatomical injuries with Injury Severity Score (ISS) > 9 or a New Injury Severity Score (NISS) ≥12.

Applying the Abbreviated Injury Scale (AIS) scores, developed by the Association for the Advancement of Automotive Medicine (AAAM), a certified registrar performs manual data coding of an annual average of about 600-700 trauma patients (Association for the Advancement of Automotive Medicine). The Head Injury Severity Scale (HISS), based on the Glasgow Coma Scale (GCS), was used to classify TBI severity as mild (GCS 14–15), moderate (GCS 9–13), or severe (GCS 3–8) (Association for the Advancement of Automotive Medicine; Stein and Spettell, 1995; Teasdale and Jennett, 1974). Alcohol use was assessed at hospital admission based on blood alcohol concentration to compare alcohol-related and non-alcohol-related TBI.

### 2.3 Variables and outcome

The study primarily focuses on the association between alcohol consumption and sex-specific differences, injury mechanisms, clinical severity, treatment variables, and patient outcome among hospitalized patients aged ≥ 60 years with TBI. As a secondary focus, the time trend was also assessed. The timeframe was stratified into five-year intervals.

### 2.4 Sex and age

In the trauma registry, demographic variables including age and sex are documented. This enables assessment of sex-specific differences in terms of alcohol consumption, mechanisms of injury, and clinical outcomes. Additionally, patients aged ≥60 years were stratified into age groups (60-69 years, 70-79 years, 80-89 years, 90+ years).

### 2.5 Mechanism of injury

Various mechanisms were documented. Road traffic accidents (RTA) and fall-related injuries constituted the two major categories. Fall injuries were further stratified into low- and high-energy injuries. Low-energy fall is defined as a fall from a height of less than 1 m. The remaining mechanisms were classified as *others*. Consequently, the relative frequency of these mechanisms in the two mean cohorts (alcohol-vs non-alcohol-related TBI) will be evaluated.

### 2.6 Blood alcohol concentration

In every trauma patient admitted to SUH blood alcohol concentration (BAC) is measured as part of the standard blood sample package. Based on the BAC the patients in this study were grouped into subclinical (0.01-0.1 g/100 ml), stimulated (0.11-0.2 g/100 ml), confused (0.21-0.3 g/100 ml), stuporous (0.31-0.4 g/100 ml) and comatose (over 0.4 g/100 ml) similar to the alcohol impairment classification of Dubowski (Jones, 2024).

### 2.7 Diagnostics and treatment

Details of diagnostic investigations, treatment, and therapeutic interventions the patients have received were also documented in the registry. The treatment was evaluated through indicators such as the length of stay in the hospital, the ICU admission and duration, and respiratory support requirements.

### 2.8 Outcome

30-day mortality and the clinical state at discharge were assessed as outcomes in the study. The discharge clinical status was evaluated based on mortality rate at discharge and injury severity using the Glasgow Outcome scale (GOS), categorized into mild, moderate, and severe, as well as permanent vegetative state (Teasdale and Jennett, 1974). Between-group comparisons were performed.

### 2.9 Statistical analysis

IBM SPSS Statistical, version 26 (IBM, New York, USA) was utilized to perform all statistical analysis in the study. The independent samples T-test was used for continuous variables, while categorical variables were analyzed using the chi-square test. A p-value<0.05 was considered statistically significant. Crude incidence rates were calculated as the total number of cases of TBI, alcohol-related, and non-alcohol-related divided by the corresponding persons at risk (≥60 years in Southwestern Norway) across the study years, expressed per 100 000 population. Population data was retrieved from Statistics Norway. Logistic regression analysis was performed to examine the association of different variables on the GOS at discharge (dichotomized into poor outcome, GOS 1-3, and good outcome, GOS 4-5). The regression model included four different variables (age, gender, alcohol use, HISS).

## 3 Results

The registry includes a total of 10.383 documented patients in the period January 1, 2004, to December 31, 2023, with 1.053 patients ≥60 years admitted with TBI. In 32 patients, the variable “alcohol use” was missing in the registry, leaving 1021 patients for analysis, 849 patients with BAC of 0.0 g/100 ml and 172 patients with BAC over 0.01 g/100 ml.

The median age for the entire cohort was 75 years (IQR 66-84). Patients with alcohol-related TBI were significantly younger with a median age of 68 years (IQR 63-73) compared to the non-alcohol-related TBI group with a median age of 77 years (IQR 67-86). Overall, males constituted 61% of the entire cohort (621 patients), with an even higher proportion observed in the alcohol-related TBI group (72% male). Table 1 presents a summary of the patients' characteristics.

**Table 1:** Patient characteristics of TBI patients 60 years and older (all, alcohol-related, and non-alcohol related) in Southwestern Norway between January 1, 2004, and December 31, 2023.

	All patients	Alcohol-related TBI	Non-alcohol-related TBI	p-value
Number of patients (%)	1021 (100)	172 (17)	849 (83)	
Age in years, median (IQR)	75 (66-84)	68 (63-73)	77 (67-86)	<0.001
Sex, no (%)				
Female	399 (39)	48 (28)	351 (41)	0.004
Male	621 (61)	124 (72)	497 (59)	
Age groups, no (%)				
60-69	376 (37)	108 (63)	268 (32)	<0.001
70-79	269 (26)	54 (31)	215 (25)	0.121
80-89	279 (27)	10 (6)	269 (32)	<0.001
90+	97 (10)	0	97 (11)	
Mechanism of injury, no (%)				
RTA	224 (22)	14 (8)	210 (25)	<0.001
Low energy fall (<1 m)	433 (42)	69 (40)	364 (43)	0.559
High energy fall	351 (34)	80 (47)	271 (32)	<0.001
Other	13 (1)	9 (5)	4 (1)	<0.001
GCS (ER), median (IQR)	14 (13-15)	14 (12-15)	15 (13-15)	<0.001
Head injury severity (ER), no (%)				
Mild	742 (73)	109 (63)	634 (75)	0.003
Moderate	148 (15)	37 (22)	111 (13)	0.006
Severe	127 (12)	26 (15)	101 (12)	0.301
Alcohol impairment, no (%)				
Subclinical	19 (2)	19 (11)	0	
Stimulated	53 (5)	53 (31)	0	
Confused	73 (7)	73 (44)	0	
Stuporous	29 (2)	19 (12)	0	
Comatose	2 (0)	2 (1)	0	
CT head				
Normal	355 (36)	79 (46)	276 (33)	0.002
Pathological	623 (63)	89 (53)	534 (67)	
ISS, median (IQR)	17 (9-22)	13 (5-18)	17 (10-24)	<0.001

IQR – Interquartile range; RTA – Road traffic accident; GCS – Glasgow Coma Scale; ER – Emergency Room; CT – Computed tomography; ISS – Injury Severity Score.

**Fig. 1:**
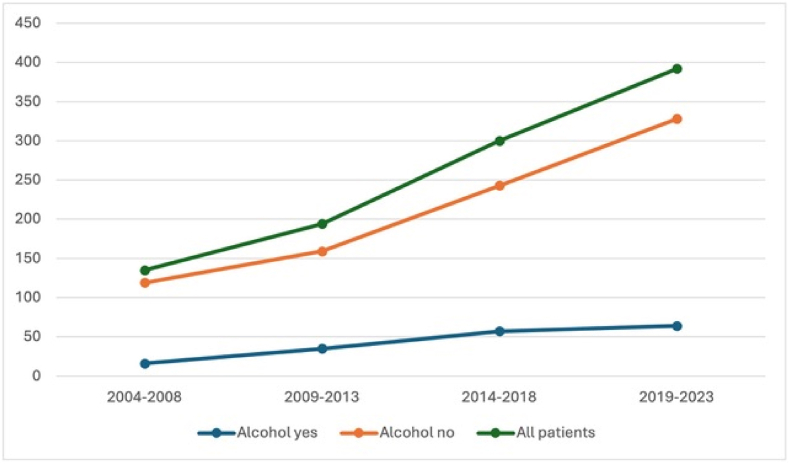
Number of hospitalized TBI patients 60 years and older (all, alcohol-related, and non-alcohol-related) in Southwestern Norway between January 1, 2004, and December 31, 2023.

**Fig. 2:**
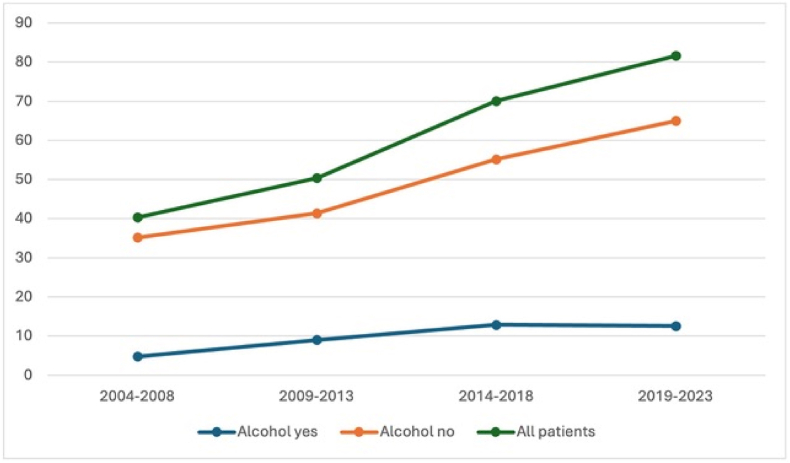
Incidence rates (per 100.000 population) of hospitalized TBI patients 60 years and older (all, alcohol-related, and non-alcohol-related) in Southwestern Norway between January 1, 2004, and December 31, 2023.

Overall, a progressive increase in hospital-registered TBI incidence was observed over time with numbers doubling throughout the 20-year study period. Both the alcohol-related and non-alcohol-related TBI groups demonstrated an upward trend, with a more pronounced rise observed in the alcohol-related TBI group. The absolute numbers of alcohol-related TBI increased fourfold, from 16 patients in the first five-year interval to 64 patients in the last one. Crude incidence increased from 40 to 81 per 100 000 population 60 years and older across the 20-year study period. Non-alcohol-related TBI doubled from 35 to 65 per 100 000 population 60 years and older, while alcohol-related TBI increased the most, almost tripled – from 5 to 13 per 100 000 population 60 years and older. Detailed temporal distribution and trend data are shown in Table 2, Fig. 1, Fig. 2.

**Table 2:** Time trends of hospitalized TBI patients 60 years and older (all, alcohol-related, and non-alcohol-related) in Southwestern Norway between January 1, 2004, and December 31, 2023, divided into four consecutive 5-year intervals.

	All patients (n = 1021)	Alcohol-related TBI (n = 172)	Non-alcohol-related TBI (n = 849)
2004-2008	135	16 (12%)	119 (88%)
2009-2013	194	35 (18%)	159 (82%)
2014-2018	300	57 (19%)	243 (81%)
2019-2023	392	64 (17%)	328 (83%)

There were no substantial differences in treatment variables between the two groups, as presented in Table 3.

**Table 3:** Treatment variables of TBI patients 60 years and older (all, alcohol-related, and non-alcohol-related) in Southwestern Norway between January 1, 2004, and December 31, 2023.

Variable	All patients (n = 1021)	Alcohol-related TBI (n = 172)	Non-alcohol-related TBI (n = 849)	p-value
Hospital LoS, median (IQR)	2 (1-6)	2 (1-4)	2 (1-6)	0.029
ICU admission, no (%)	363 (36)	62 (36)	301 (35)	1.000
ICU LoS, median (IQR)	1 (1-5)	2 (1-5)	1 (1-5)	0.317
Ventilator treatment, no (%)	89 (9)	21 (12)	68 (8)	0.764
Ventilator LoS, median (IQR)	2 (1-5)	2 (1-5)	2 (1-5)	0.915

LoS – the length of stay; ICU – Intensive Care Unit.

As presented in Table 4, the 30-day mortality rate was higher in the non-alcohol-related TBI group (17% versus 8%, p 0.027). Among the patients who died in the alcohol-related TBI group, the majority of deaths (10 of 14) were attributed to TBI. In contrast, fewer TBI-related deaths were observed in the non-alcohol group (82 of 146 patients), and a greater proportion of deaths occurred after discharge (30 of 146). At discharge, 46% of all patients had moderate disability. The non-alcohol group showed a slightly poorer outcome, with a higher proportion experiencing severe disability (15% versus 9%), and three patients in this group were in a permanent vegetative state.

**Table 4:** Outcome of TBI patients 60 years and older (all, alcohol-related, and non-alcohol-related) in Southwestern Norway between January 1, 2004, and December 31, 2023.

Variable	All patients (n = 1021)	Alcohol-related TBI (n = 172)	Non-alcohol-related TBI (n = 849)	p-value
Mortality 30 days, no (%)	160 (16)	14 (8)	146 (17)	0.027
due to TBI	92 (9)	10 (6)	82 (10)	0.108
GOS at discharge, no (%)				
Good recovery	269 (26)	53 (31)	216 (25)	0.172
Moderate disability	471 (46)	91 (53)	380 (45)	0.061
Severe disability	143 (14)	15 (9)	128 (15)	0.039
Persistent vegetative state	3 (0)	0	3 (0)	1.000
Death	129 (13)	13 (8)	116 (14)	0.038

In multivariable logistic regression analysis, increasing age (OR 1.04 per year, p < 0.001), male sex (OR 1.44, p = 0.039) and higher head injury severity were independently associated with poor outcome (Table 5), whereas alcohol was not associated with poorer outcome.

**Table 5:** Binary logistic regression analysis of TBI patients 60 years and older (all, alcohol-related, and non-alcohol-related) in Southwestern Norway between January 1, 2004, and December 31, 2023.

Variable.	OR	95% CI for OR	p-value
Age	1.04	1.03-1.06	<0.001
Sex (male)	1.44	1.02-2.04	0.039
Alcohol use (yes)	0.37	0.22-0.62	<0.001
HISS (moderate versus mild)	5.27	3.51-7.90	<0.001
HISS (severe versus mild)	22.56	13.74-37.05	<0.001

## 4 Discussion

### 4.1 Principal findings

The results of this study showed an increasing number of older patients hospitalized with alcohol related TBI over the study period of two decades. Alcohol-related TBI accounted for 17% of the study population, indicating a considerable proportion in this patient population over 60 years of age. Male patients were overrepresented within the entire cohort, with an even higher proportion observed in the alcohol-related TBI group.

The primary objective of this study was to evaluate the association between alcohol and TBI and the temporal trends in the older patient population. Comparable trends have been reported in studies with overlapping research focuses.

### 4.2 Temporal trends in TBI and alcohol consumption

The findings indicate that TBI among older patients demonstrated an increasing trend. A 1.5-fold increase in total TBI cases was observed, and the absolute number remained higher in the non-alcohol group. However, the most pronounced increase was observed within the alcohol-related TBI group of older male adults. The absolute number in the last five-year interval of the study period was approximately fourfold higher than in the initial interval. Corresponding increasing results were observed in crude incidence rates, with the most pronounced increase in alcohol-related TBI. The true incidence is likely higher, considering all mild TBI patients managed outside the hospital, pre-hospital deaths, or delayed clinical presentations, such as chronic subdural hematoma symptoms, making them not captured in the setting of an acute care trauma registry (Braden et al., 2024; Pantelatos et al., 2023; Uno, 2023).

A systematic analysis for the Global Burden of Disease Study demonstrated an increasing TBI incidence in older adults. The incidence increased by 4.4% from 1990 to 2016 within the ≥60 years old population globally. Male patients showed the greatest increase in incidence, with age-standardized incidence rates increased by 4.4% versus 1.8% for female in that period (Global, 2019). A previous Norwegian study, which focused on time trends of TBI in older patients, reported similar findings as the current study, in which both consultations and hospital admissions due to TBI among older Norwegians doubled multiple times between 2006 and 2023 (Sandvik, 2024). Several underlying determinants may account for the observed upward trend in TBI incidence among older individuals. Life expectancy in Norway has risen over the past two decades (World Health Organization), resulting in a larger older patient population and consequently an increase in TBI incidence. While older individuals today maintain higher physical capacity, concomitant factors, such as polypharmacy and comorbidities, are elevating exposure to injury risk (Pfortmueller et al., 2014). In parallel, this higher incidence may partially reflect increased health awareness, improved diagnostics, and registration of TBI cases.

The incidence of alcohol consumption is showing an increasing trend as well. In Norway, the proportion of current drinkers increased from 60% in 1985 to 83% in 2019 in the population aged ≥60 years (Bye and Moan, 2020). Similar trends have been observed in the USA. The prevalence of past-month binge alcohol use among individuals aged ≥65 years significantly increased from 8.1% in 2005-06 to 9% in 2013-14, representing an 11.1% relative increase (Han et al., 2017). Although the absolute estimates differ between our study and these studies, the overarching message is that TBI is increasing alongside a rising alcohol consumption incidence in older adults as a conclusion.

### 4.3 Clinical characteristics and outcomes of alcohol-related TBI

Injury mechanisms varied between groups as well. High-fall injuries were more frequent in the alcohol group, suggesting potentially increased higher-risk behavior associated with alcohol use. At the same time, low-fall injuries were slightly more common within the non-alcohol group, likely reflecting an older and more comorbid population within this group (Aarsland et al., 2024). Even though low-energy falls dominated, a major part of the study population (those over 60 years) sustained high-energy falls, which is somewhat surprising.

Clinically, patients in the alcohol-related TBI group demonstrated marginally lower GCS, and a higher proportion of patients sustained severe head injuries and were admitted to the ICU compared to the non-alcohol group. Nevertheless, the GCS difference was relatively small.

Despite these findings, the median ISS score was significantly lower in the alcohol-related TBI group with lower prevalence of abnormal CT findings as well, and 30-day mortality was higher in the non-alcohol-related TBI group (17% versus 8%). In addition, patients in the alcohol-related TBI group experienced better clinical outcomes at discharge, based on the Glasgow Outcome Scale (GOS). These findings may be explained by a significantly higher median age in the non-alcohol-related TBI group; increasing age is a risk factor for poor outcome after TBI (Roe et al., 2013).

Despite lower GCS scores, it is challenging to determine that patients with alcohol-related TBI experienced poorer outcomes based on GCS values alone. The GCS may be confounded by the depressive effects of alcohol consumption (Pina and Marco, 2024). Accordingly, GCS alone cannot conclusively determine whether alcohol was associated with increased injury severity. Conversely, a previous study reported that alcohol did not significantly reduce GCS in trauma populations (Stuke et al., 2007). Necessary diagnostic evaluation and treatment may be delayed due to misattribution of reduced GCS to alcohol intoxication (Stuke et al., 2007). Although these findings are challenging to interpret, a possible hypothesis is that alcohol users constitute a healthier baseline population, whereas the other group could have higher baseline vulnerability. Additionally, the patients in the alcohol group were younger on average, which may support the hypothesis of a relatively healthier baseline status in the alcohol group (Stelander et al., 2023).

Accordingly, the determinants of clinical outcome are complex. To further evaluate predictors of outcome, multivariable logistic regression analysis was applied. Interestingly, the findings support the hypothesis that many variables influence the outcome, where age, sex and higher injury severity were independent predictors for poorer outcome. In contrast, alcohol exposure was not independently associated with poorer outcome in the adjusted analysis. Nevertheless, these findings should be interpreted with caution, as number alcohol-related TBI cases is increasing - raising the possibility that alcohol use is predisposing to TBI, which may consequently affect clinical outcomes (Weil et al., 2018).

### 4.4 Sex differences

Globally, approximately 7% of injury-related deaths in 2019 were directly attributed to alcohol. Males (90%) accounted for most alcohol-attributable injury deaths (Chikritzhs and Livingston, 2021). A systematic review conducted in Europe further indicates that male patients are at higher risk of sustaining TBI. For instance, 80% of reported TBI cases in Ireland were male, an even higher proportion compared to our findings of 61% (Brazinova et al., 2021). Although the gender gap in alcohol consumption is narrowing, males remain overrepresented (White et al., 2015). These results highlight the significant role of alcohol in the epidemiology of TBI. Moreover, the gender gap remains pronounced, despite alcohol use among older women has risen recently (Brazinova et al., 2021), male patients are still overrepresented in both alcohol-related trauma and TBI populations.

### 4.5 Clinical implications and future perspectives

The triad of aging, alcohol consumption, and TBI represents an essential future challenge, requiring focused efforts and evidence-based interventions to reduce both human and socio-economic burdens. The findings in this study indicate that the older patient population is growing, alcohol consumption is rising, and the incidence of TBI is increasing. Despite these trends, limited research has directly examined this relationship and the underlying causes. Hence, broader representative studies are required to further elucidate this concerning issue, as well as investigating etiological factors. In conjunction, new strategies and efforts should be developed and implemented. Shifting the treatment paradigm toward earlier preventive interventions, enhanced diagnostics, optimized treatment, and modernized rehabilitation supported by new resources, including artificial intelligence (De la Torre et al., 2025).

## 5 Strengths and limitations

The findings in the study are based on data from a thoroughly maintained trauma registry, which is a reliable and valid data source. While the registry was first established in 2003, complete inclusion protocols were fully standardized from 2004 onwards, by systematic identification and documentation of trauma patients by certified registrars. Consequently, enhancing the data consistency and stability of the registry. In addition to a long observation period, which minimizes short-term fluctuations and enhances the robustness and generalizability of the findings.

We cannot rule out a small degree of selection bias since we only have data on patients who were actually admitted to hospital. Many patients with isolated minor or mild TBI are handled by primary health care institutions and not registered in the hospital's trauma registry. Consequently, the data presents incidence of hospital-registered TBI and may therefore underestimate the true population burden of TBI.

## 6 Conclusion

The study shows that TBI in older patients has increased over time and is mostly caused by fall injuries. Alcohol-related TBI within this patient group has also increased over the past two decades. Male older patients were more often suffering from TBI, with an even higher proportion observed in relation to alcohol consumption.

## Ethics approval and consent to participate

Ethics approval was granted by The Regional Committee for Medical and Health Research Ethics of Western Norway (REK143902/2020).

## Declaration of generative AI and AI-assisted technologies in the manuscript preparation process

Grammarly was used for grammar checking. No patient data was provided to any AI tools. After using these tools, the authors reviewed and edited the content as necessary and take full responsibility for the content of the published article.

## Funding

This research did not receive any specific grant from funding agencies in the public, commercial, or not-for-profit sectors.

## Declaration of competing interest

The authors declare no conflicts of interest.
